# Large positive linear magnetoresistance in the two-dimensional *t*_*2g*_ electron gas at the EuO/SrTiO_3_ interface

**DOI:** 10.1038/s41598-018-26017-z

**Published:** 2018-05-16

**Authors:** Kristy J. Kormondy, Lingyuan Gao, Xiang Li, Sirong Lu, Agham B. Posadas, Shida Shen, Maxim Tsoi, Martha R. McCartney, David J. Smith, Jianshi Zhou, Leonid L. Lev, Marius-Adrian Husanu, Vladimir N. Strocov, Alexander A. Demkov

**Affiliations:** 1 Department of Physics, The University of Texas at Austin, Austin, Texas 78712 USA; 2 Materials Science and Engineering Program/Mechanical Engineering, University of Texas at Austin, Austin, Texas 78712 USA; 3 School of Engineering for Matter, Transport and Energy, Arizona State University, Tempe, AZ 85287 USA; 4 Department of Physics, Arizona State University, Tempe, Arizona 85287 USA; 5 Paul Scherrer Institute, Swiss Light Source, CH-5232 Villigen PSI, Switzerland; 6 National Research Centre “Kurchatov Institute”, 1 Akademika Kurchatova pl., 123182 Moscow, Russia; 7 National Institute of Materials Physics, 405A Atomistilor Str., 077125 Magurele, Romania

## Abstract

The development of novel nano-oxide spintronic devices would benefit greatly from interfacing with emergent phenomena at oxide interfaces. In this paper, we integrate highly spin-split ferromagnetic semiconductor EuO onto perovskite SrTiO_3_ (001). A careful deposition of Eu metal by molecular beam epitaxy results in EuO growth via oxygen out-diffusion from SrTiO_3_. This in turn leaves behind a highly conductive interfacial layer through generation of oxygen vacancies. Below the Curie temperature of 70 K of EuO, this spin-polarized two-dimensional *t*_*2g*_ electron gas at the EuO/SrTiO_3_ interface displays very large positive linear magnetoresistance (MR). Soft x-ray angle-resolved photoemission spectroscopy (SX-ARPES) reveals the *t*_*2g*_ nature of the carriers. First principles calculations strongly suggest that Zeeman splitting, caused by proximity magnetism and oxygen vacancies in SrTiO_3_, is responsible for the MR. This system offers an as-yet-unexplored route to pursue proximity-induced effects in the oxide two-dimensional *t*_*2g*_ electron gas.

## Introduction

The high mobility two-dimensional *t*_*2g*_ electron gas (2DEG) present at oxide/oxide interfaces is currently under intense investigation^1^. In particular, different types of magnetism have been observed in the oxide 2DEG^2^ providing a richness of physical phenomena ripe for being exploited in novel oxide devices^3^ as in the recent successful demonstration of a spin-polarized 2DEG in engineered LAO/STO-based heterostructures using EuTiO_3_^4^. Thin films of perovskite oxides exhibit superconductivity^5^ and colossal magnetoresistance^6^, magnetism^7^, ferroelectricity^8^ and multiferroicity^9^, piezoelectricity^10^, and thermoelectricity^11^. On the other hand, rock salt EuO, a nearly ideal Heisenberg ferromagnet, boasts a large saturation magnetic moment of 7 μ_B_, with corresponding unprecedented 0.6 eV spin-splitting of the conduction 5*d* band^12^. Thus EuO is ideal for spin filtering^13^, and is considered as a strong candidate for future spintronic applications^13^. There have also been proposals to combine EuO with Al thin films^14^, graphene layers^15^ and MoTe_2_^16^ layers, to induce ferromagnetism in these systems by proximity effects.

Thus, it is of great fundamental interest to search for novel physical phenomena at the interface of ferromagnetic semiconductor EuO with other complex oxides. Recent first-principles calculations predict a fully spin-polarized 2DEG at the LaAlO_3_/EuO interface due to electrostatic doping from the polar oxide^17,18^. However, from a thermodynamic perspective, creation of the heterostructure suggested by Lee *et al*.^18^ is rather difficult. EuO is not stable under ambient conditions^19^, much less the oxygen-rich high-temperature environment necessary for the deposition of crystalline LaAlO_3_^20^. However, among the various mechanisms for the oxide 2DEG formation, one viable approach involves tailoring an interface between SrTiO_3_ (STO) and oxides with large negative enthalpy of formation such as EuO^21^ to stabilize a confined conducting layer of SrTiO_3−δ_^22,23^. This approach offers an elegant route of bringing together strong ferromagnetism and *t*_*2g*_ 2DEG at the oxide interface.

Here we demonstrate very large positive linear magnetoresistance of the *t*_*2g*_ 2DEG at the interface of epitaxial EuO/STO, for EuO films in the thickness range of ~5–10 nm grown by molecular beam epitaxy (MBE). Growth is achieved by depositing Eu metal onto STO (001) without oxygen in ultra-high vacuum. The x-ray diffraction (XRD) and scanning transmission electron microscopy (STEM) show that epitaxy on the TiO_2_-terminated STO plane results in rock salt EuO (Eu^2+^). The crystalline EuO thin films are ferromagnetic below the Curie temperature of 70 K with a saturation moment ~6.3 μ_B_/Eu.as demonstrated by superconducting quantum interference device (SQUID) magnetometer. Low-temperature transport measurements were performed in the physical property measurement system (PPMS). These EuO/STO heterostructures display temperature-dependent linear positive magnetoresistance below the Curie temperature. X-ray photoemission spectroscopy (XPS) shows a valence band offset of 2 eV and closely aligned conduction bands. Density functional theory (DFT) analysis based on the XRD/STEM-derived structural model provides a consistent picture of the band alignment, magnetic state of EuO, and electronic structure of the oxygen-deficient conductive layer formed in STO. Using soft x-ray angle-resolved photoemission spectroscopy (SX-ARPES), we elucidate the *d*_*xy*_ t_2g_ character of the low dimensional electron system. The carriers reside at the STO side of the EuO/STO interface, which conclusively demonstrates symmetry breaking due to carrier confinement and thus the existence of the 2DEG. First principles calculations show that magnetoresistance is proportional to spin polarization that is linear in field due to the Zeeman effect. Combining these results, we uncover the role of the spin-polarized oxygen vacancy as the origin of the linear positive magnetoresistance stemming from the ferromagnetism of Eu^2+^ magnetic moments in proximity to the confined oxygen-deficient conductive layer.

## Results

### Sample Preparation and Characterization

Since EuO is highly sensitive to oxygen pressure and tends to form Eu_2_O_3_, special care is needed to ensure proper stoichiometry. In general, EuO epitaxy must be carefully controlled with regards to temperature, deposition rate, and oxygen pressure to preserve the Eu^2+^ oxidation state: metallic Eu^0^ has a low sticking coefficient^24^, while over-oxidized Eu^3+^ is paramagnetic^25^. Here, we build upon the previous multi-metal study^21^ of oxygen scavenging from SrTiO_3_ and demonstrate that it is possible to crystallize stoichiometric EuO by depositing Eu metal onto SrTiO_3_ (001) under ultra-high vacuum, where oxygen is provided only by the substrate. Details of the growth window were investigated by *in situ* x-ray photoelectron spectroscopy, as summarized in Supplementary Fig. S1. For *ex situ* characterization, a capping layer of 2-nm Al_2_O_3_ was deposited directly after growth. For observations by scanning transmission electron microscopy (STEM), a 10-nm Ti capping layer was deposited to protect the surface from oxidation during STEM sample cross-sectioning for viewing along the SrTiO_3_ [100]/EuO [110] projection.

**Figure 1:**
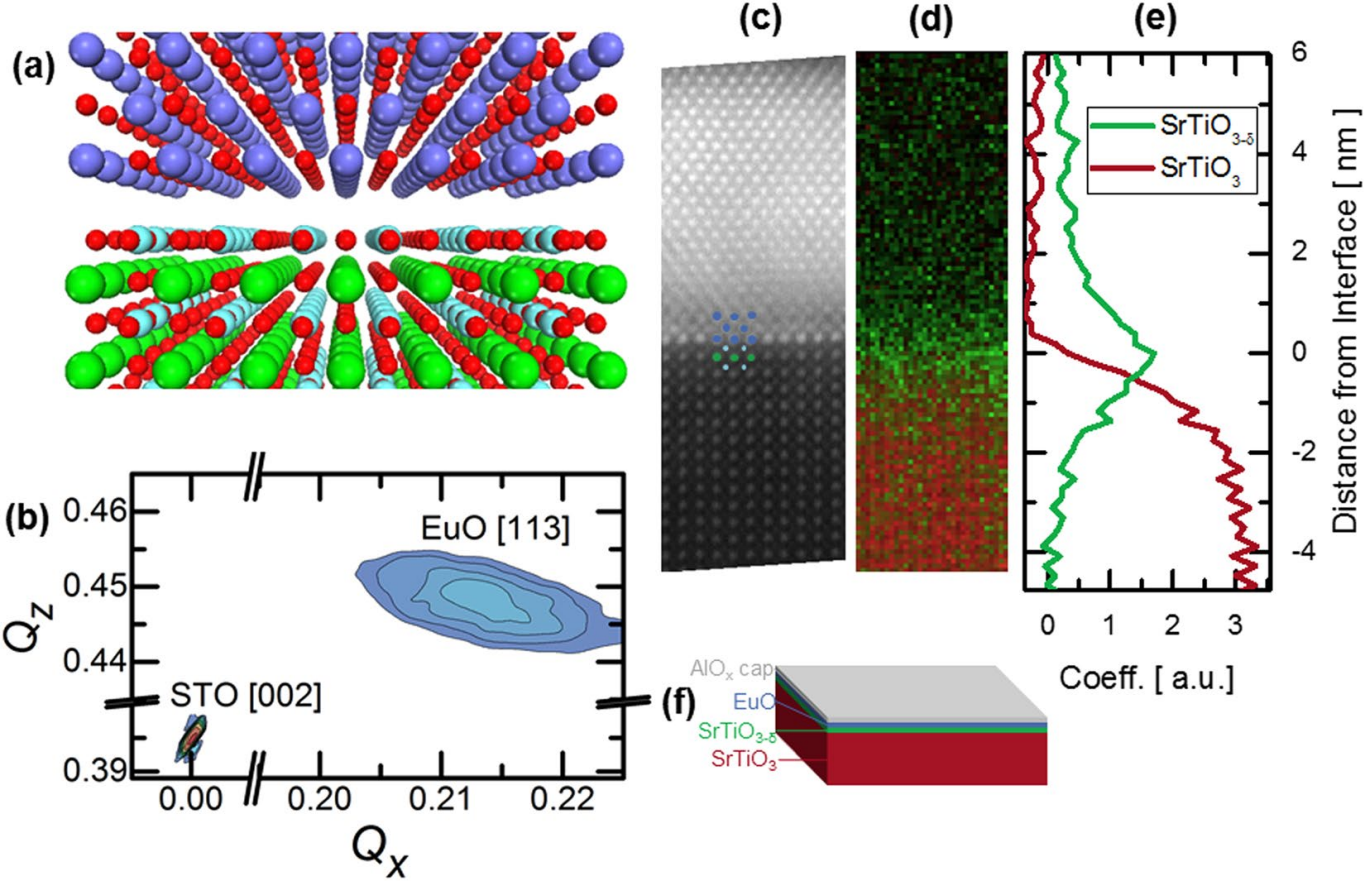
Epitaxy of EuO on SrTiO_3_ (001). (**a**) Atomic model of the rocksalt (top)/perovskite (bottom) heterointerface. (**b**) Reciprocal space map of the STO (002) and EuO (113) peaks for 7 nm EuO on STO. The EuO rocksalt unit cell is rotated 45° with respect to the surface unit cell of the perovskite. (**c**) High-angle annular-dark-field scanning transmission electron microscopy [100]-projection image of the EuO/STO interface. (**d**) Corresponding false color map shows a distribution map from the Ti L-edge fit (SrTiO_3_, red; SrTiO_3−δ_, green). (**e**) Ti-L coefficient as a function of position shows a sharp peak at the interface. (**f**) Overall schematic, including the bulk STO substrate (red), layer of STO with oxygen vacancies (green), EuO film (blue), and capping layer (gray). Not to scale.

The EuO films crystallize in the rock-salt structure (Space group *Fm3m*^26^) and the primary unit cell axis is rotated by 45° with respect to the unit cell axis of the substrate surface to minimize lattice mismatch (22% down to ~7%). The films are fully relaxed, as shown schematically in Fig. 1(a) and in the reciprocal space map in Fig. 1(b). Additional x-ray diffraction results are provided in Supplementary Fig. S2. Lattice parameters extracted from the in-plane and out-of-plane scans are 0.513 and 0.515 nm, respectively.

Aberration-corrected STEM imaging as well as atomically-resolved column-by-column energy-loss near-edge structure (ELNES) analysis, were used to determine the sample structure and to map out changes in the Ti and Eu oxidation states across the EuO/SrTiO_3_ interface using the method reported previously^27^. Published spectra were used as references for the Eu-N edge^23^. As shown in the high-angle annular-dark-field image of the EuO/SrTiO_3_ interface in Fig. 1(c), the films are epitaxial with defects in the first few layers. Furthermore, from the edge-fitting of the Ti-L energy-loss near-edge fine-structure^28^, false color maps as shown in Fig. 1(d) reveal the distribution of oxygen vacancies associated with partially reduced Ti^3+^ at the interface. This result is qualitatively in good agreement with x-ray photoelectron spectroscopy results reported for Eu metal on SrTiO_3_ by Posadas *et al*.^21^. Complementary Eu-N edge data confirming Eu^2+^ oxidation state are given in Supplementary Fig. S3. It has also been shown theoretically that due to the large dielectric constant, of SrTiO_3_, the 2DEG can spread across 50 unit cells in the low density region (n < 10^14^ *cm*^−2^). In the high density region (n > 5 × 10^14^ *cm*^−2^), which is relevant here, the 2DEG is mostly confined within a few unit cells, though the tail may still be quite long^29^. The proximity of EuO to the confined SrTiO_3−δ_ conducting layer is shown in the heterostructure cross-section in Fig. 1(e).

### Electrical Characterization

**Figure 2:**
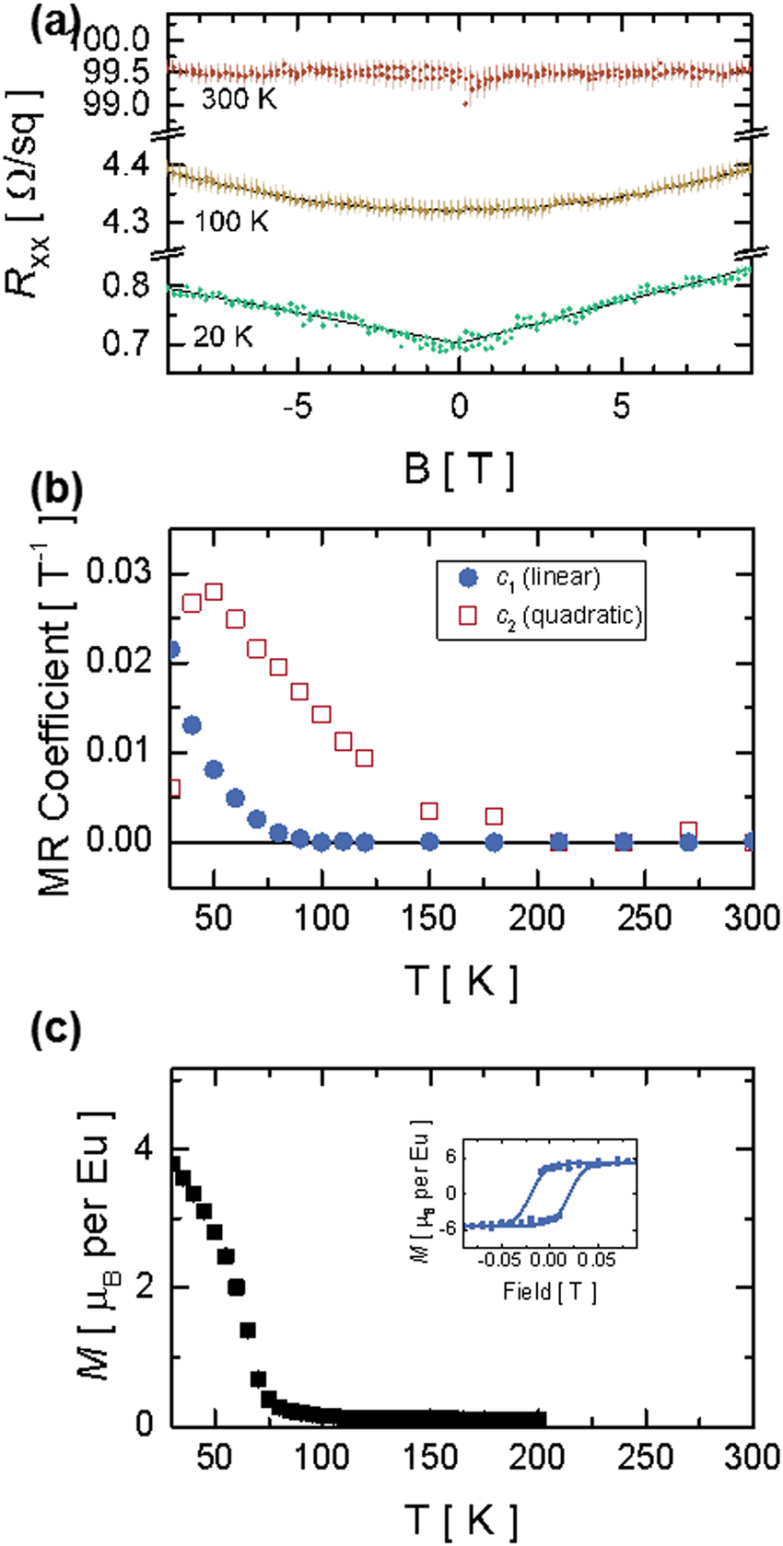
(**a**) Magnetoresistance (MR) data measured in a perpendicular magnetic field at 20 K, 100 K, and 300 K. Solid lines indicate fits to the data. The MR increases linearly with the magnetic field at 20 K, quadratically at 100 K, and is field-independent at room temperature. (**b**) Linear *c*_1_ and quadratic *c*_2_ MR fit coefficients for the same film as a function of temperature. (**c**) Field-cooled magnetization *M* of a similar 7-nm EuO film as a function of temperature at constant in-plane magnetic field of 0.01 T. Inset: corresponding magnetization loop measured at 10 K.

The EuO film exhibits a paramagnetic to ferromagnetic transition with decreasing temperature as seen in Fig. 2(c), which shows the field-cooled magnetization of a 7-nm EuO film as a function of temperature. Curie-Weiss fitting to this data gives a Curie temperature of *T*_C_ ~ 70 K and an effective moment of ~6.3 *μ*_B_/Eu. From the magnetization loops measured at 10 K with magnetic fields applied in the plane of the film [see inset to Fig. 2 (c)] we extract a coercive field ~0.02 T and remnant magnetization ~4.3 *μ*_B_/Eu. These are essentially the values for bulk EuO.

Measurements of the sheet resistance *R*_S_ for 7-nm EuO/STO over a temperature range from 2–300 K reveal metallic behavior (Supplementary Fig. S4(a)). Hall measurements indicate high sheet carrier densities on the order of 10^16^ cm^−2^ (Supplementary Fig. S4(b)). Figure 2(a) shows four-probe magnetoresistance *R*_S_(*B*) measurements for a 7-nm EuO film in a perpendicular magnetic field. *R*_S_ increases linearly with the magnetic field at 20 K and quadratically at 100 K. Solid lines indicate fits to the data of the form,

1 $${R}_{s}(B)={R}_{s}(0)\times [{c}_{1}|B|+{({c}_{2}B)}^{2}],$$

where *c*_1_ and *c*_2_ are the linear and quadratic fit coefficients, respectively, shown in Fig. 2(b) as a function of temperature. The quadratic magnetoresistance component is present below ~150 K, while the linear component emerges below ~80 K. The magnetoresistance (MR), defined as,

2 $$MR=\frac{{R}_{s}(B)-{R}_{s}(0)}{{R}_{s}(0)},$$

decreases rapidly as the measurement temperature increases, and is essentially zero at room temperature as shown in Fig. 2(a).

The quadratic MR component can be attributed to the ordinary magnetoresistance found in normal metals, stemming from the Lorentz force. On the other hand, the much more interesting linear MR needs special consideration. Since its emergence coincides with the Curie temperature (~70 K), we ascribe the origin of the positive linear MR to a Zeeman split of the 2DEG electronic structure^30^ induced by magnetic ordering of oxygen vacancies in the top STO layer, as DFT modelling illustrates below.

### First-principles Calculations

**Figure 3:**
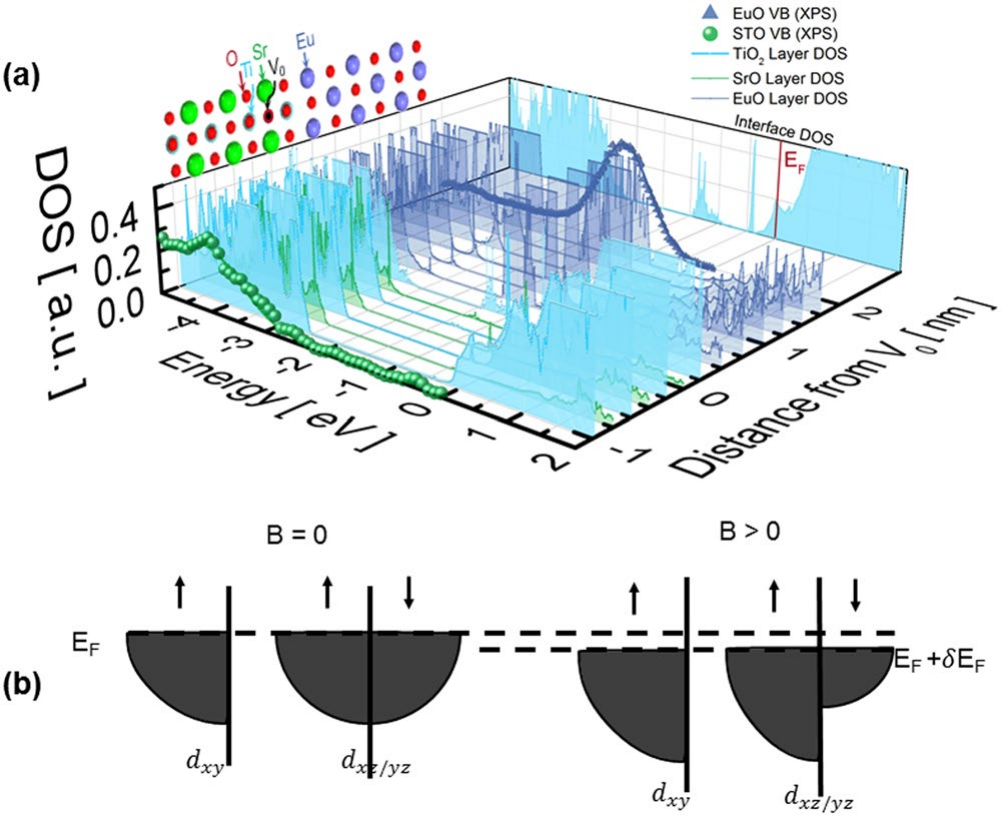
(**a**) Spin-up density of states (DOS) projected onto atomic planes across the EuO/STO simulation cell, with an oxygen vacancy (V_0_) at the SrO layer below the interface. The oxygen vacancy state can be seen at −0.4 eV. This state decays within ~0.2 nm from the interface. The theoretical valence band (VB) shapes and valence band offset are in good agreement with experimental data, also shown. (**b**) Schematic illustration of Zeeman shift.

For these EuO/SrTiO_3−δ_/SrTiO_3_ (001) heterostructures, the band alignment is crucial in determining the spatial extent of the conducting SrTiO_3−δ_ layer and therefore the magnitude of the wave function overlap, which is the origin of the exchange proximity interaction^31^. The density of states (DOS) and valence band offset at the EuO/SrTiO_3−δ_ interface calculated from first principles are in good agreement with the x-ray photoelectron spectroscopy (XPS) data shown in Fig. 3 (the details of the first-principles calculations are given in Supplementary Methods). Band offset measurement by XPS is described in Supplementary Note 1 and Supplementary Fig. S6. Figure 3 shows the simulation cell with one oxygen vacancy in the sub-interface SrO layer and the corresponding DOS projected onto atomic planes across the heterostructure. The interface structure in the calculations is kept consistent with STEM images recorded in the [110] projection (Supplementary Fig. S5).

A sharp, spin-up Eu 4 *f* state forms the valence band edge of EuO and is fully spin-polarized, with a magnetic moment of 7 *μ*_*B*_/Eu. This is in good agreement with studies of ferromagnetism in bulk EuO^32^. A localized impurity state emerges 0.4 eV below the Fermi level. This state, residing on two Ti atoms adjacent to the vacancy, has an *e*_*g*_ orbital character mixed with *p*_*z*_ due to lifting of the local cubic symmetry induced by a vacancy^33^. Importantly, the in-gap state is singly-occupied and polarized with its spin aligned with the Eu ion above the interface. The impurity state decays quickly into both EuO and SrTiO_3_, with the evanescent states present up to 2 layers away from the vacancy plane. The decay length is 0.19 nm and 0.18 nm in EuO and STO, respectively, consistent with the complex band structure^34^.

Inspecting carriers in the conduction band of SrTiO_3_, we note that most itinerant electrons are located on the SrTiO_3_ side, and the delocalized Ti *t*_*2g*_ states are occupied by the second electron of the vacancy. Recent theoretical studies^35,36^ suggested that the vacancy-induced localized state can trap at most one electron, while the second electron occupies the conduction band when correlation effects are taken into account. Interestingly, experiment indicates that at the LaAlO_3_/SrTiO_3_ interface, oxygen vacancies result in a local magnetic moment on Ti^3+^ that couples antiferromagnetically with the 2DEG^37^. From the orbital-projected DOS (Supplementary Fig. S7), we find that the itinerant occupied states at the interface are purely *d*_*xy*_ orbitals, but become mainly *d*_*xz*_/*d*_*yz*_ away from the interface. The split-off *d*_*xy*_ band has also been reported at the LaAlO_3_/SrTiO_3_ interface and attributed to orbital reconstruction due to symmetry lowering^38^. It is worth noting that the occupied *d*_*xy*_ state at our interface is spin-split by ~0.3 eV and thus the interfacial carriers are fully spin-polarized in the same way as the vacancy-induced in-gap state and the Eu 4f state. When considering the exact same heterostructure without a vacancy but with an extra electron (introduced artificially), we still see spin polarization in the *d*_*xy*_ band at the interface^39^. This suggests that the interface carriers are influenced by the 7 *μ*_*B*_ moment on the neighbouring Eu ions and the spin-polarization of the 2DEG is caused by the proximity effect.

To explain the positive linear MR we consider the Zeeman shift of spin-split *d*_*xy*_ bands. Below the critical temperature *T*_c_, EuO becomes ferromagnetic and, as suggested by calculation (Supplementary Fig. S7), carriers at the interface (*d*_*xy*_) are spin-polarized while those in deeper layers (*d*_*xz/yz*_) remain nonmagnetic. With an external field, the spin-polarized *d*_*xy*_ band and the initially-nonmagnetic spin-up band shift downward while the initially-nonmagnetic spin-down band shifts upward, as shown in Fig. 3(b). As detailed in Supplementary Note 2, in the presence of strong scattering at the interface the MR is positive and linear in magnetic field^30^. The enhanced scattering is due to FM alignment of vacancy-related moments and *d*_*xy*_ 2DEG.

In principle, there are several other mechanisms that might account for positive linear magnetoresistance (LMR) such as quantum electron-electron interference^40^, sample inhomogeneity^41^, and electron correlation^42,43^. However, following the work of Lee *et al*.^40^ and Gerber *et al*.^44^, the calculated quantum correction is several orders of magnitude smaller as compared with our measurement. A quantum correlation model^42,43^ (for fields of ~10T) is based on the assumption that the energy spectrum is gapless and linear and requires the electron density on the order of 10^18^ *cm*^−3^. None of these conditions holds in our case. The inhomogeneity mechanism results in the positive LMR over a broad temperature range and LMR is independent of carrier density^41^. In our case the positive LMR is present only below the EuO Curie temperature T_c_ and is quadratic above T_c_, similar to conventional semiconductors. It would be too much of a coincidence for the inhomogeneity to introduce LMR exactly at T_c_ of EuO. Hence, we believe the Zeeman shift of spin-split bands offers the most natural explanation.

### Photoemission

**Figure 4:**
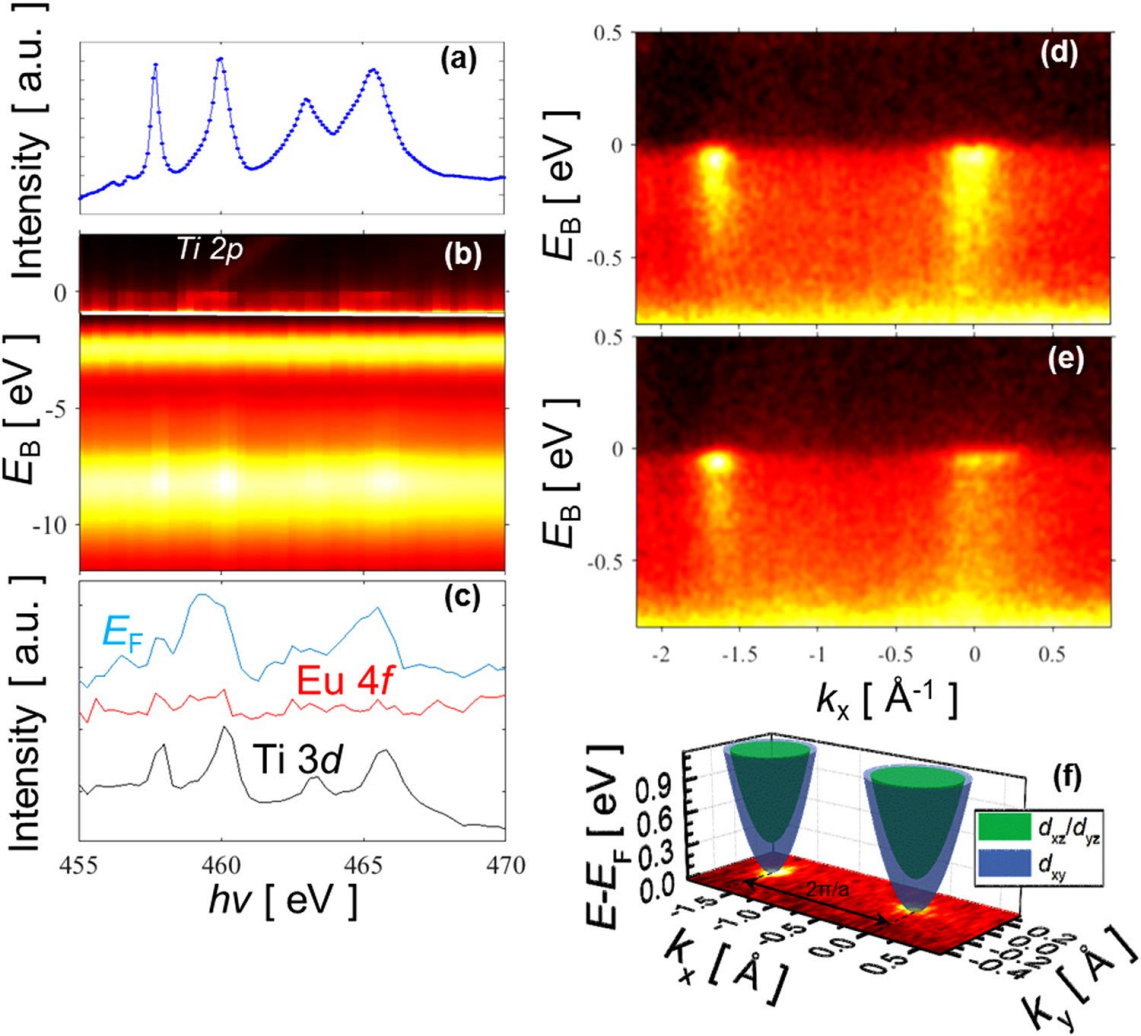
Resonant soft-X-ray ARPES of 2-nm EuO/STO heterointerface through the Ti *L*-edge. (**a**) XAS spectrum. (**b**) Resonant photoemission from the valence band as a function of excitation energy. Intensity in the near-*E*_F_ region is scaled up by ~30. (**c**) Resonant intensity for constant *E*_B_ in the valence band, Eu level and 2DEG. The valence band and 2DEG signals follow the Ti *L*-edge XAS spectrum that confirms their Ti-derived character. (**d**,**e**) SX-ARPES images at *hv* = 460.3 and 466 eV enhancing the *d*_xy_- and *d*_yz_-derived states, respectively. The intensity waterfalls reveal the polaronic nature of the interface charge carriers. (**f**) Fermi surface of the interface states measured at *hv* = 466 eV.

To visualize the band structure of the EuO/SrTiO_3_ interface resolved in electron momentum **k**, we used soft-x-ray angle-resolved photoemission spectroscopy (SX-ARPES). Spectral response of the buried interface states was boosted using resonant photoexcitation at the Ti 2*p* absorption edge. In Fig. 4, we present the experimental X-ray absorption spectra and resonant (angle integrated) photoemission intensity across the Ti 2*p* edge. The latter embeds the Ti *t*_2g_ derived 2DEG signal at *E*_F_, the Eu 4 *f* feature around *E*_B_ ~ −2.5 eV, and the O 2*p* derived valence band states of EuO and SrTiO_3_ below. Figure 4(c) shows the photoemission intensity variations in the corresponding *E*_B_-regions. The 2DEG and valence band response resonates near the Ti absorption peaks. This confirms, respectively, the Ti 3*d* origin of the 2DEG and the hybridization of the O 2p states with Ti, similar to the paradigm LaAlO_3_/SrTiO_3_ interface^45,46^. On the other hand, the Eu 4 *f* response shows no correlation with the Ti 2*p* absorption, which indicates vanishing hybridization between the Eu 4 *f* and Ti 3*d* states. Furthermore, similar resonant data at the Eu 3*d* absorption edge (Supplementary Fig. S8) shows no sign of any significant admixture of Eu 4 *f* states in the 2DEG. This indicates that the 2DEG in the EuO/SrTiO_3_ heterostructure resides on the SrTiO_3_ side of the interface, in good agreement with density functional calculations.

Photoelectron images visualizing electron dispersions *E*(**k**) in the 2DEG are shown in Fig. 4(d) for *hv* = 460.3 eV emphasizing the Ti *d*_xy_ states localized near the interface, and Fig. 4(e) for 466 eV emphasizing the Ti *d*_yz_/*d*_xz_ states more extended into the SrTiO_3_ bulk^46^. This is consistent with our DFT calculations in the previous section. The EuO/SrTiO_3_ interface shows much smaller band filling compared to the LaAlO_3_/SrTiO_3_ case^46^ that is manifested by small Fermi vector *k*_F_ of the heavy *d*_yz_ band in Fig. 4(e). The waterfalls going from the band dispersions down in *E*_B_ is a hallmark of all SrTiO_3_-based systems that signal the polaronic nature of the charge carriers with their characteristic peak-dip-hump spectral response involving electron coupling to the LO3 phonon^46,47^. Furthermore, significantly smaller intensity of the EuO/SrTiO_3_ interface bands compared to LaAlO_3_/SrTiO_3_ may indicate a larger fraction of the non-conducting interfacial phase^48,49^

Finally, Fig. 4(f) shows the Fermi surface formed by the interface electrons. This was measured at *hv* = 466 eV to emphasize external contours formed by the ellipsoidal Ti *d*_yz_/*d*_xz_ sheets. As expected from the experimental *E*(**k**) dispersions, the Fermi surface is nevertheless dominated by the circular *d*_xy_ derived electron pocket with only small filling of the *d*_yz_/*d*_xz_ sheets compared to the LaAlO_3_/SrTiO_3_ case^46^. Therefore, the overall electron density in our case has stronger interface localization compared to LaAlO_3_/SrTiO_3_ interface.

## Conclusions

In summary, we have discovered large linear positive MR in the EuO/SrTiO_3−δ_/SrTiO_3_ heterostructure grown by depositing Eu metal onto SrTiO_3_ (001). Such deposition enables crystallization of stoichiometric highly-spin-polarized EuO semiconductor in close proximity to a highly conductive interfacial layer of oxygen-deficient SrTiO_3−δ_. The EuO films are ferromagnetic with a Curie temperature of 70 K and the interfacial 2DEG displays linear positive MR below the EuO Curie temperature. Using density functional theory, we demonstrate a defect-driven spin-polarized 2DEG at the interface, with the *t*_*2g*_ character of the low-dimensional electron system confirmed by resonant SX-ARPES. Combining these results, we uncover the role of the spin-polarized oxygen vacancy states as the origin of the linear positive MR, suggesting a path towards developing novel nano-oxide spintronic devices based on strong proximity effects.

## Methods

### Film deposition

SrTiO_3_ (001) substrates with dimensions 5 mm × 5 mm × 0.5 mm (commercially available with TiO_2_-termination by HF etching from Crystec) were degreased in acetone, isopropanol, deionized water, and UV ozone. The samples were then introduced into a customized DCA 600 MBE system with a base pressure of 6 × 10^–10^ Torr. More details of the experimental system can be found elsewhere^50^. All substrates were outgassed in the MBE chamber at 700° for 10 min under ultra-high vacuum (UHV). The substrate temperature was measured by a thermocouple (calibrated by pyrometer measurement of a silicon substrate) in proximity to the substrate heater.

The substrate temperature during EuO deposition was fixed at 200 °C. Eu metal flux evaporated from an effusion cell was calibrated to a metal deposition rate of ~0.36 nm/min as measured by a quartz crystal microbalance. Molecular oxygen was introduced at a partial pressure varied between 1 × 10^−10^ to 1 × 10^−8^ Torr. The samples were monitored during growth *in situ* by RHEED. After film deposition, the films were capped with ~1.4 nm aluminum metal to form ~2-nm alumina upon exposure to ambient conditions for *ex situ* electrical and magnetic characterization.

### Sample Characterization

XPS measurements were performed *in situ* using a VG Scienta R3000 electron energy analyzer with monochromatic Al Kα radiation (hν = 1486.6 eV).

To electrically contact the capped EuO/STO interface, four indium contacts were placed on corners of each sample in a van der Pauw geometry. Measurements were performed with a Physical Property Measurement System (PPMS) from Quantum Design capable of applying a ±9 T magnetic field. The magnetization measurements of a 7-nm-thick EuO (001) film were carried out as a function of temperature under field-cooled conditions at a constant magnetic field of 0.01 T oriented in-plane with a SQUID magnetometer (Quantum Design).

### Density functional theory

First-principles calculations based on density functional theory (DFT) were performed using generalized gradient approximation^51^ (GGA) for the projector augmented wave pseudopotentials^52^, as implemented in the Vienna Ab-Initio Simulation Package code^53^. For Sr, Ti, Eu and O, 4*s*^2^4*p*^6^5*s*^2^, 3*s*^2^3*p*^6^4*s*^2^3*d*^2^, 5*s*^2^5*p*^6^4*f* ^7^6*s*^2^ and 2*s*^2^2*p*^4^ are included, respectively. The plane-wave cutoff energy was 600 eV. To correct the on-site Coulomb interaction and consider the correlation effect in SrTiO_v_, we adopted Dudarev’s rotationally invariant approach^54^ adding a Hubbard U term (GGA + U). Typical values U_f_ = 5.0 eV and U_d_ = 5 eV, J_d_ = 0. 64 eV were used for Eu localized 4 *f* orbitals and Ti 3*d* orbitals, respectively. We employed symmetric (EuO)_3_(STO)_6_(EuO)_3_ supercell geometry with vacuum region thicker than 1 nm to prevent interaction between adjacent slabs. The interface was TiO_2_-terminated and Eu atoms were on top of hollow positions in TiO_2_ plane, as continuation of Sr atoms. Lattice parameter a_STO_ = 0.395 nm was used and EuO layers were rotated by 45° to match the lattice constant (8% tensile strain on EuO layers). For creation of a single vacancy, an O atom was removed at the sub-interface SrO layer in a 2 × 2 slab. All atom positions were fully relaxed until residual forces were less than 0.2 eV nm^−1^. The Brillouin zone was sampled with 4 × 4 × 1 Monkhorst-Pack *k*-point grids^55^.

### Soft-X-ray ARPES experiments

These experiments have been carried out at the SX-ARPES end station^56^ of the ADRESS beamline^57^ at the Swiss Light Source (Paul Scherrer Institute, Switzerland). Circularly polarized X-rays were incident on the sample at a grazing angle of 20° to increase photoelectron yield from the buried EuO/SrTiO_3_ interface. The sample was cooled down to 12 K to quench the thermal effects reducing the coherent **k**-resolved spectral component at high photoexcitation energies^58^. The combined (beamline and analyzer) energy resolution was ~100 meV. The SX-ARPES resonant measurements at the Ti *L*-edge were complemented by X-ray absorption spectroscopy (XAS) measurements in total electron yield.

With an intense photon flux of about 2 × 10^13^ ph/sec delivered by the ADRESS beamline into a spot of ~30 × 74 μm^2^ on the sample, the SX-ARPES spectra significantly depended on the X-ray irradiation as evidenced by gradual increase of spectral intensity^59^. This can be seen in the time evolution of the SX-ARPES images of 2DEG presented in Supplementary Fig. S9. The irradiation causes two different effects going on in parallel. First, it recovers the oxygen vacancies in STO largely quenched by oxygen out-diffusion from the STO bulk. One of the vacant electrons left by the vacancy stays localized at the Ti^3+^ ion, and another is injected into the mobile 2DEG^36,49^. Second, after several weeks of shelf life before the SX-ARPES experiment a significant fraction of the EuO layer is oxidized to Eu_2_O_3_. Similar to vacancy creation in STO, the irradiation creates them in Eu_2_O_3_ or, in other words, reducing the sesquioxide to EuO-like oxygen stoichiometry. This opens another path for vacancy creation in STO by scavenging of oxygen by Eu^21^. Therefore, a noteworthy part of our SX-ARPES experiment was a partial recovery of the original sample stoichiometry under X-ray irradiation. This partial recovery of the EuO layer is illustrated in Supplementary Fig. S10 that shows a dramatic increase of the Eu^2+^ fraction in the spot on the sample exposed to X-rays till saturation after ~30 min. Of key importance in this recovery of the EuO/STO system under irradiation, is that that the formation of vacancy-based 2DEG adopts a phase-separation scenario, where the conducting 2DEG paddles are embedded in otherwise insulating STO^49^. Whereas the integral area of these paddles increases with the concentration of vacancies and extension of their distribution towards the STO bulk, the local electronic structure inside the paddles stays unchanged. Our SX-ARPES spectra measured under a saturating dose of X-ray irradiation are therefore representative, to a large degree, of the authentic EuO/STO samples with spin-polarized 2DEG.

## Electronic supplementary material

Supplementary Information
