# Supplementary material for: Large positive linear magnetoresistance in the two-dimensional t2g electron gas at the EuO/SrTiO3 interface
Source: Sci Rep. 2018 May 16;8:7721. doi: 10.1038/s41598-018-26017-z (PMC5955958; doi:10.1038/s41598-018-26017-z)
Supplement: Supplementary file 1 — Supplementary Information [file 41598_2018_26017_MOESM1_ESM.pdf]

Supplementary Information for Scientific Reports

Large positive linear magnetoresistance in the two-dimensional t_{2g} electron gas at the EuO/$SrTiO_3$ interface

Kristy J. Kormondy,[1] Lingyuan Gao,[1] Xiang Li,[2] Sirong Lu,[3] Agham B. Posadas,[1] Shida Shen,[1] Maxim Tsoi,[1] Martha R. McCartney,[4] David J. Smith,[4] Jianshi Zhou,[2] Leonid L. Lev,[5,6] Marius-Adrian Husanu,[5,7] Vladimir N. Strocov,[5] and Alexander A. Demkov[1a)]

[1]*Department of Physics, The University of Texas at Austin, Austin, Texas, 78712, USA*
[2]*Materials Science and Engineering Program/Mechanical Engineering, University of Texas at Austin, Austin, Texas, 78712, USA*
[3]*School of Engineering for Matter, Transport and Energy, Arizona State University, Tempe, AZ 85287, USA*
[4]*Department of Physics, Arizona State University, Tempe, Arizona, 85287, USA*
[5]*Paul Scherrer Institute, Swiss Light Source, CH-5232 Villigen PSI, Switzerland*
[6]*National Research Centre "Kurchatov Institute", 1 Akademika Kurchatova pl., 123182 Moscow, Russia*
[7]*National Institute of Materials Physics, 405A Atomistilor Str. 077125, Magurele, Romania*

Supplementary Note 1

Supplementary Figure S6(a) displays XPS valence band spectra for the bare STO substrate (blue shading) and 10-nm-thick EuO film (red shading). Band offsets were calculated by both core-level[1] and valence band[2,3] spectroscopy. The valence band maximum (E_{VBM}) for each material was calculated using the linear extrapolation method.[4] By comparing energy offsets between the Ti 2p, Eu 3d, and valence band edge positions (as summarized in Table 1) using

$$VBO_{CL} = \left(E_{Eu3d} - E_{VBM}\right)_{EuO} - \left(E_{Ti2p} - E_{VBM}\right)_{STO} - \left(E_{Eu3d} - E_{Ti2p}\right)_{EuO/STO} \quad \textbf{(1)}$$

we calculate a VBO_{CL} of 2.44 eV.

a) demkov@physics.utexas.edu

The VBO can also be determined directly from the valence band spectra.[2,3] For a heterostructure of 1 nm EuO/STO a contribution from the substrate is visible along with an attenuated contribution from the substrate (Supplementary Figure S6 (a), black open circles). We simulated a fit (purple solid line) to the heterostructure by scaling and offsetting the pure valence band spectra measured for the STO substrate and thick EuO film. This method uses an "all at once" fit minimizing χ^2 by the Levenberg-Marquardt algorithm implemented in Igor Pro software (WaveMetrics, Lake Oswego, OR). The difference between the measured and simulated spectra is also plotted (black line). Using this method, we calculate VBO_{sim} = 2.26 eV, in good agreement with the offset calculated above by the core levels. The band profile is illustrated in Supplementary Figure S6(b).

Supplementary Note 2

Our calculations follow Onose et al.'s work[5], which is based on Boltzmann transport theory. In the present case, we have one interface (IF) $\boldsymbol{d^{\uparrow}}_{\boldsymbol{xy}}$ band, and three unpolarized t_{2g} bands for deep layers (we call them bulk t_{2g} states). All these states are confined along the z direction. From *ab-initio* calculations, the IF band is found to be split down by Δ compared with the other bands and Δ is about 0.15 eV. Thus, we can write the energy near the $\boldsymbol{\Gamma}$ point for these bands[6]:

$$\varepsilon = \begin{cases} \frac{\hbar^2}{2m_L}{k_x}^2 + \frac{\hbar^2}{2m_L}{k_y}^2 - \Delta & IF\ d^{\uparrow}{}_{xy} \\ \frac{\hbar^2}{2m_L}{k_x}^2 + \frac{\hbar^2}{2m_L}{k_y}^2 & bulk\ d_{xy} \\ \frac{\hbar^2}{2m_L}{k_x}^2 + \frac{\hbar^2}{2m_H}{k_y}^2 & bulk\ d_{xz} \\ \frac{\hbar^2}{2m_H}{k_x}^2 + \frac{\hbar^2}{2m_L}{k_y}^2 & bulk\ d_{yz} \end{cases}$$

Here, m_L stands for light mass while m_h stands for heavy mass. According to ARPES experiments[7], $m_L^* \approx 0.7m_e$, $m_H^* \approx 10{\sim}20m_e$.

Without considering the spin degree of freedom, we can get the density of states (DOS) for the confined t_{2g} states:

$$D = \begin{cases} \frac{m_L}{2\pi\hbar^2} & d_{xy} \\ \frac{\sqrt{m_L m_H}}{2\pi\hbar^2} & d_{xz} \\ \frac{\sqrt{m_L m_H}}{2\pi\hbar^2} & d_{yz} \end{cases}$$

Taking the 3D conductivity tensor as a reference[8], we get the 2D conductivity tensor for the three t_{2g} states:

$$\sigma^{xx} = \begin{cases} \frac{e^2\tau}{2}\frac{\varepsilon_F}{\pi\hbar^2} & d_{xy} \\ \frac{e^2\tau}{2}\frac{\varepsilon_F}{\pi\hbar^2}\sqrt{\frac{m_H}{m_L}} & d_{xz} \\ \frac{e^2\tau}{2}\frac{\varepsilon_F}{\pi\hbar^2}\sqrt{\frac{m_L}{m_H}} & d_{yz} \end{cases}$$

From this, we can see that the contribution of the d_{yz} state to σ^{xx} is very small compared that of the d_{xz} state.

With an external magnetic field H, the majority band is shifted down and the minority band is shifted up. In our case, to first order, the change in the Fermi energy $\delta\varepsilon_F$ could be obtained by number conservation:

$$\delta\varepsilon_F \approx \frac{1}{2} g\mu_B H \frac{\sum_{t_{2g}} D^{\downarrow}(\varepsilon_F) - \sum_{t_{2g}} D^{\uparrow}(\varepsilon_F) - D^{\uparrow}{}_{IF}(\varepsilon_F + \Delta)}{\sum_{t_{2g}} D^{\downarrow}(\varepsilon_F) + \sum_{t_{2g}} D^{\uparrow}(\varepsilon_F) + D^{\uparrow}{}_{IF}(\varepsilon_F + \Delta)},$$

where $\sum_{t_{2g}} D(\varepsilon_F)$ takes the summation of DOS over all three bulk t_{2g} bands at ε_F and $D^{\uparrow}{}_{IF}(\varepsilon_F + \Delta)$ refers to the DOS of the IF $\boldsymbol{d}^{\uparrow}{}_{\boldsymbol{xy}}$ band.

The conductivity without external magnetic field can be written as:

$$\sigma^{xx}(H=0) = {\sigma_{IF_{xy(\uparrow)}}}^{xx} + {\sigma_{bulk\ d_{xy}(\uparrow)}}^{xx} + {\sigma_{bulk\ d_{xy}(\downarrow)}}^{xx} + {\sigma_{bulk\ d_{xz}(\uparrow)}}^{xx} + {\sigma_{bulk\ d_{xz}(\downarrow)}}^{xx} + {\sigma_{bulk\ d_{yz}(\uparrow)}}^{xx} + {\sigma_{bulk\ d_{yz}(\downarrow)}}^{xx} = \frac{e^2\tau_{IF}}{2}\frac{\varepsilon_F+\Delta}{\pi\hbar^2} + 2\times\frac{e^2\tau_{bulk\ d_{xy}}}{2}\frac{\varepsilon_F}{\pi\hbar^2} + 2\times\frac{e^2\tau_{bulk\ d_{xz}}}{2}\frac{\varepsilon_F}{\pi\hbar^2}\sqrt{\frac{m_H}{m_L}} + 2\times\frac{e^2\tau_{bulk\ d_{yz}}}{2}\frac{\varepsilon_F}{\pi\hbar^2}\sqrt{\frac{m_L}{m_H}}$$

Under an external magnetic field H, the conductivity becomes:

$$\sigma^{xx}(H \neq 0) = \frac{e^2\tau_{IF}}{2}\frac{\varepsilon_F + \delta\varepsilon_F + \Delta + \frac{1}{2}g\mu_B H}{\pi\hbar^2} + \frac{e^2\tau_{bulk\ d_{xy}}}{2}\frac{\varepsilon_F + \delta\varepsilon_F + \frac{1}{2}g\mu_B H}{\pi\hbar^2}$$
$$+ \frac{e^2\tau_{bulk\ d_{xy}}}{2}\frac{\varepsilon_F + \delta\varepsilon_F - \frac{1}{2}g\mu_B H}{\pi\hbar^2} + \frac{e^2\tau_{bulk\ d_{xz}}}{2}\frac{\varepsilon_F + \delta\varepsilon_F + \frac{1}{2}g\mu_B H}{\pi\hbar^2}\sqrt{\frac{m_H}{m_L}}$$
$$+ \frac{e^2\tau_{bulk\ d_{xz}}}{2}\frac{\varepsilon_F + \delta\varepsilon_F - \frac{1}{2}g\mu_B H}{\pi\hbar^2}\sqrt{\frac{m_H}{m_L}}$$
$$+ \frac{e^2\tau_{bulk\ d_{yz}}}{2}\frac{\varepsilon_F + \delta\varepsilon_F + \frac{1}{2}g\mu_B H}{\pi\hbar^2}\sqrt{\frac{m_L}{m_H}}$$
$$+ \frac{e^2\tau_{bulk\ d_{yz}}}{2}\frac{\varepsilon_F + \delta\varepsilon_F - \frac{1}{2}g\mu_B H}{\pi\hbar^2}\sqrt{\frac{m_L}{m_H}}$$

By taking the difference and plugging in what we had before, we get:

$$\sigma^{xx}(H) - \sigma^{xx}(0) = \frac{e^2\frac{1}{2}g\mu_B H}{2}\frac{\frac{2\left((\tau_{IF}-\tau_{bulk\ d_{xy}})\frac{m_L}{2\pi\hbar^2}+(\tau_{IF}-\tau_{bulk\ d_{xz}}+\tau_{IF}-\frac{m_L}{m_H}\tau_{bulk\ d_{yz}})\frac{\sqrt{m_L m_H}}{2\pi\hbar^2}\right)}{3\frac{m_L}{2\pi\hbar^2}+4\frac{\sqrt{m_L m_H}}{2\pi\hbar^2}}}{\pi\hbar^2}$$

The change of magnetoresistance is:

$$\rho(H) - \rho(0) = \frac{1}{\sigma(H)} - \frac{1}{\sigma(0)} = \frac{1}{\sigma(0)}\left(\frac{1}{\left(1 + \frac{\sigma(H)-\sigma(0)}{\sigma(0)}\right)} - 1\right) \approx -\frac{1}{\sigma(0)^2}\left(\sigma(H) - \sigma(0)\right)$$
$$= -\frac{e^2 g\mu_B H}{2\sigma(0)^2}\frac{\frac{\left((\tau_{IF}-\tau_{bulk\ d_{xy}})\frac{m_L}{2\pi\hbar^2}+(\tau_{IF}-\tau_{bulk\ d_{xz}}+\tau_{IF}-\frac{m_L}{m_H}\tau_{bulk\ d_{yz}})\frac{\sqrt{m_L m_H}}{2\pi\hbar^2}\right)}{3\frac{m_L}{2\pi\hbar^2}+4\frac{\sqrt{m_L m_H}}{2\pi\hbar^2}}}{\pi\hbar^2}$$

To get a positive linear magnetoresistance (LMR), the sufficient conditions are:

$$\tau_{IF} < \tau_{bulk\ d_{xy}},\ \ 2\tau_{IF} < \tau_{bulk\ d_{xz}} + \frac{m_L}{m_H}\tau_{bulk\ d_{yz}} \approx \tau_{bulk\ d_{xz}}$$

Considering strong scattering at the interface, it is highly possible that the above conditions could be satisfied. Therefore, we argue that the Zeeman effect is the likely origin of positive LMR in our system.

Supplementary Figures

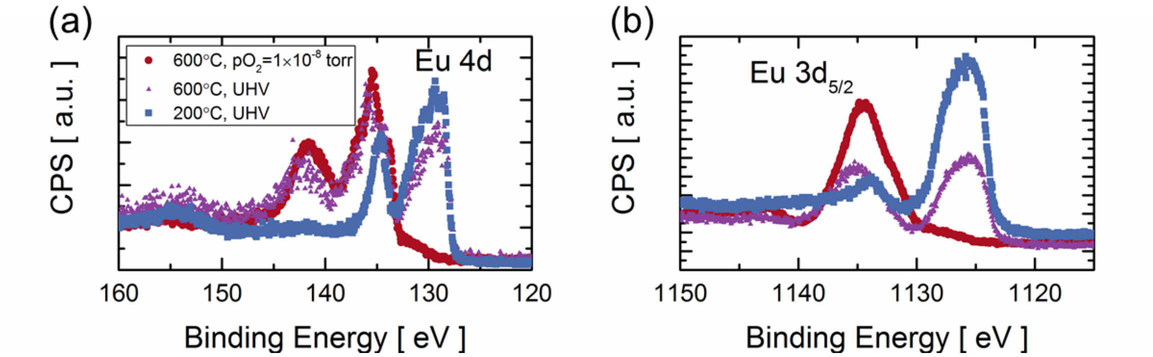

Supplementary Figure S1: XPS Eu (a) 4*d* and (b) 3*d* core level measurements for varied substrate temperature and oxygen partial pressure.

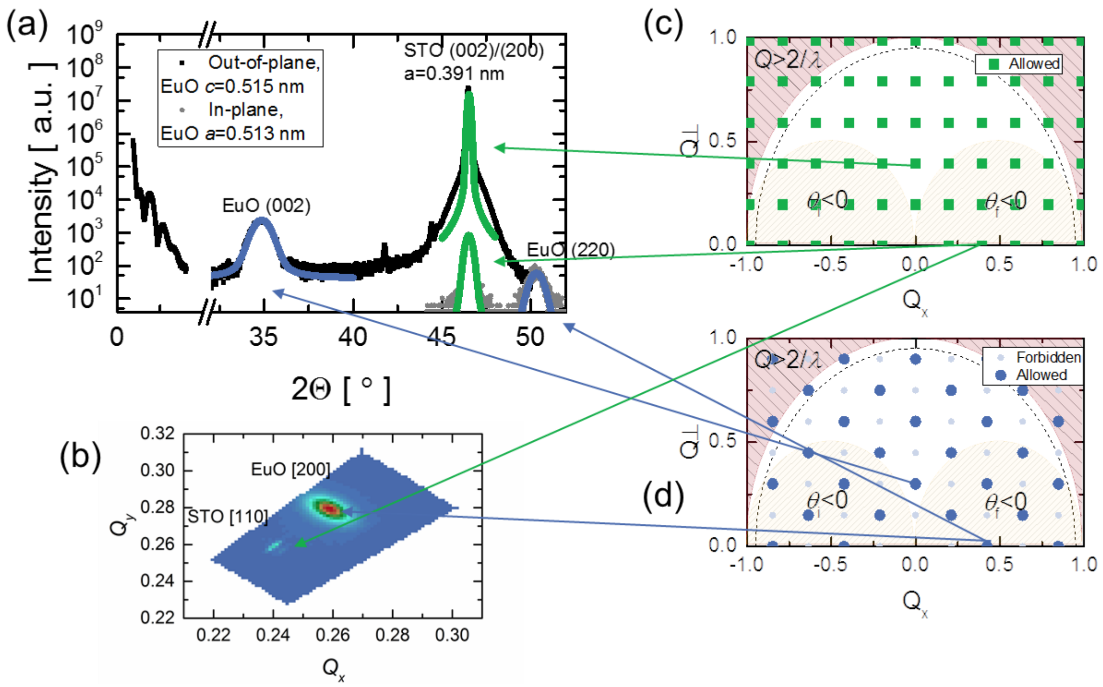

Supplementary Figure S2: (a) X-ray diffraction coupled scans with corresponding (b) in-plane reciprocal space map and positions in reciprocal space for (c) STO and (b) EuO with a 45° rotation of the surface unit cell.

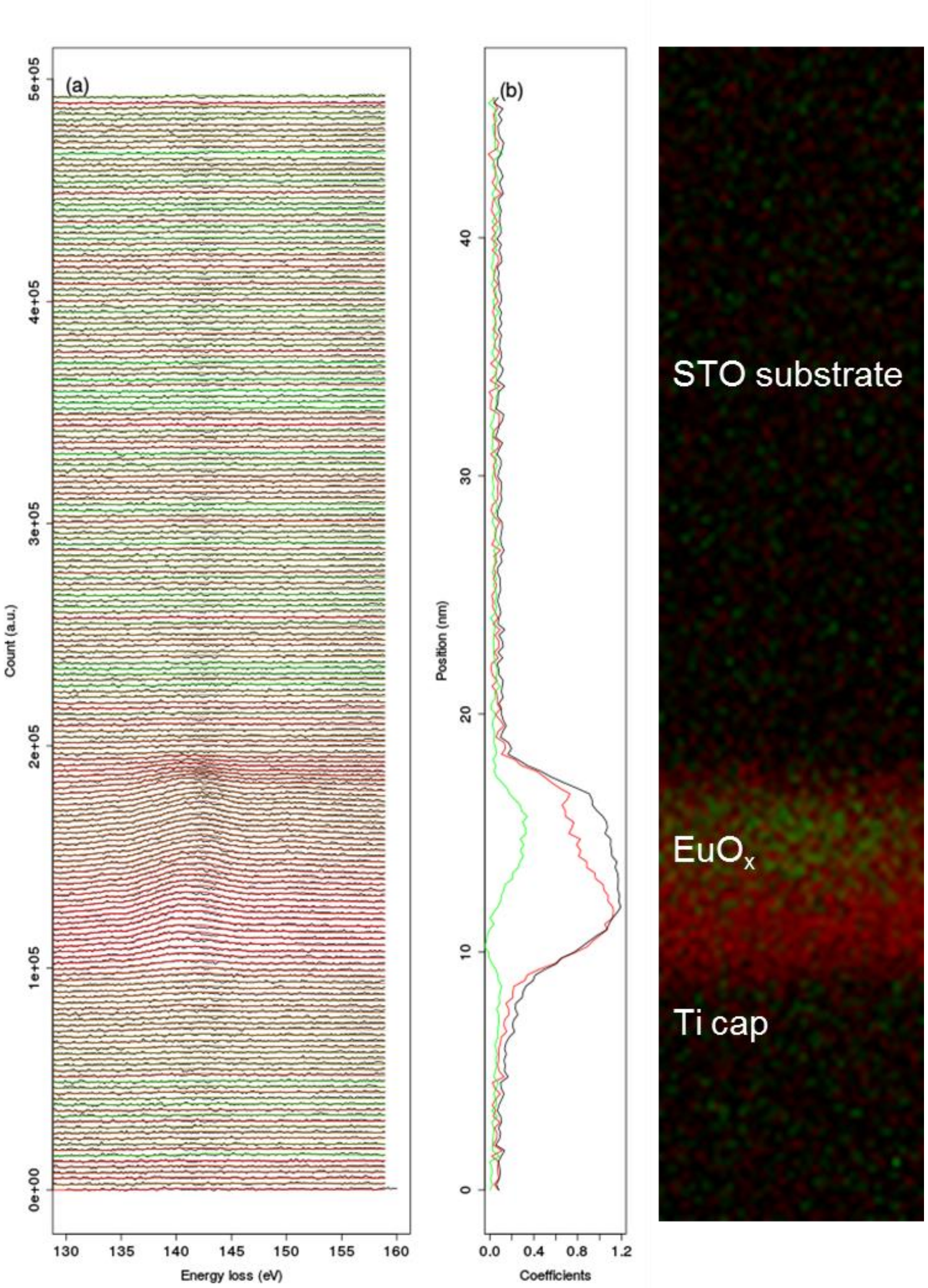

Supplementary Figure S3: MLLS fitting of layer-resolved EELS spectra. Red is EuO and Green is Eu_2O_3. Only a small amount of Eu^{3+} is present in the disordered region. (Reference spectra taken from Mairoser et al.[9]).

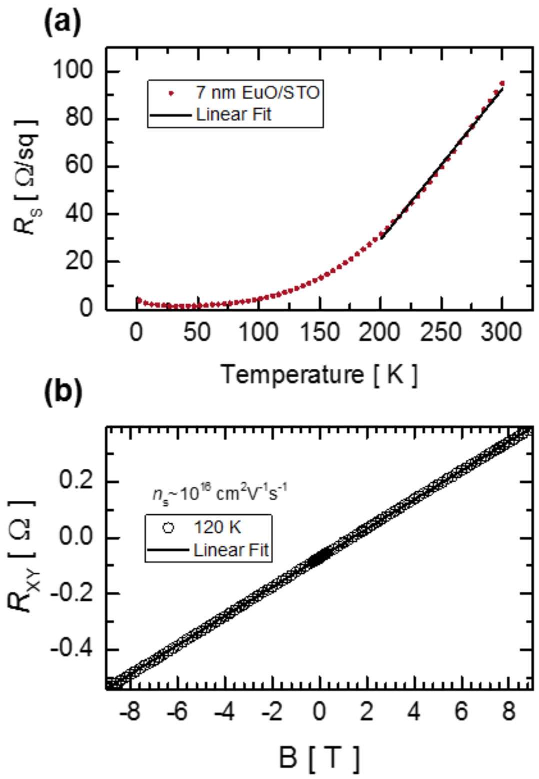

Supplementary Figure S4: (a) Sheet resistance for a 7-nm EuO film as a function of temperature. (b) Hall resistance R_{XY} for a 7-nm EuO film at 120 K. Solid lines indicate linear fits.

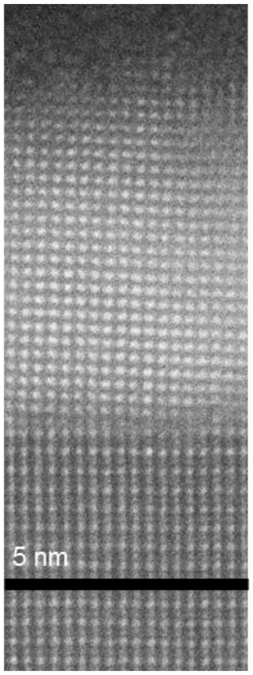

Supplementary Figure S5: High-angle annular dark-field scanning transmission electron microscopy [110]-projection image of the EuO/STO interface.

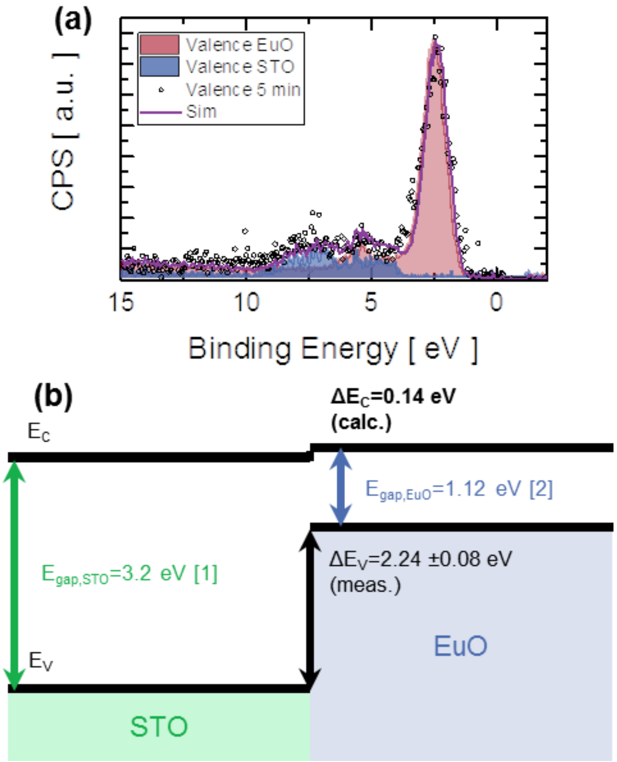

Supplementary Figure S6: (a) To determine the valence band offset, the spectra for pure EuO (red shading) and STO (blue shading) were scaled, offset, and added together to create a simulated fit (purple line) to the measured valence band spectra for a heterostructure of 1 nm EuO on STO (black open circles). (b) Schematic of band alignment.

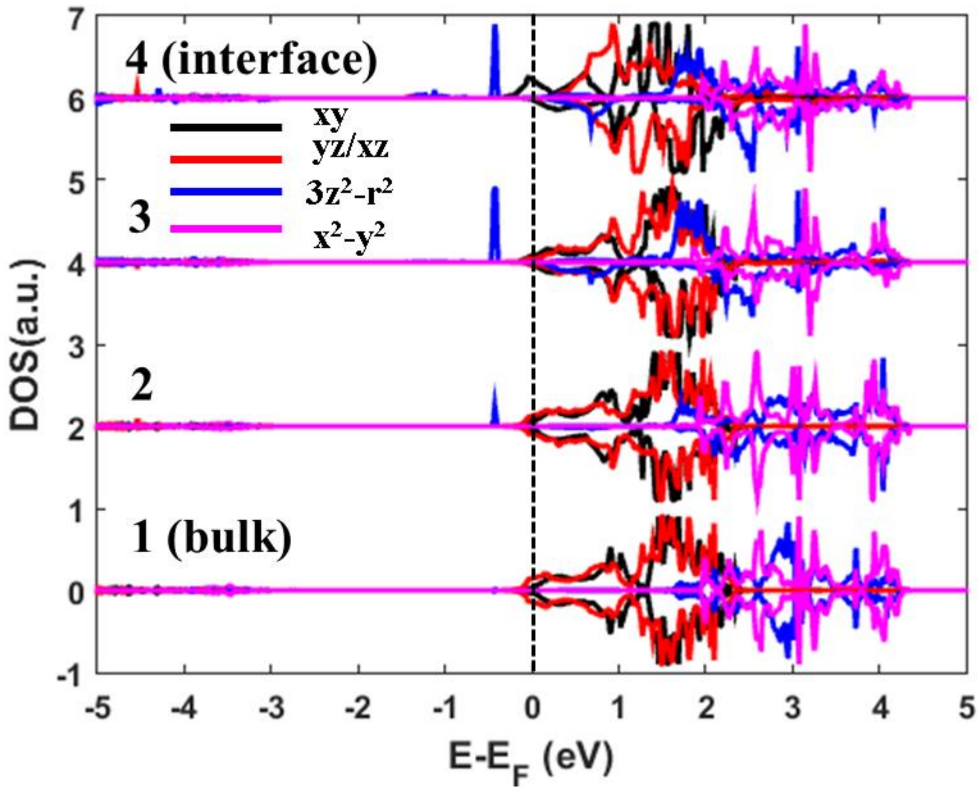

Supplementary Figure S7: The partial density of states projected onto Ti ions at each layer. Interface TiO_2 layer is at top and middle bulk TiO_2 layer is at bottom. d_{xy} orbitals are marked as black line. d_{xz} and d_{yz} orbitals are marked as red line. $\boldsymbol{d_{3z^2-r^2}}$ orbitals are marked as blue and $\boldsymbol{d_{x^2-y^2}}$ orbitals are marked as magenta.

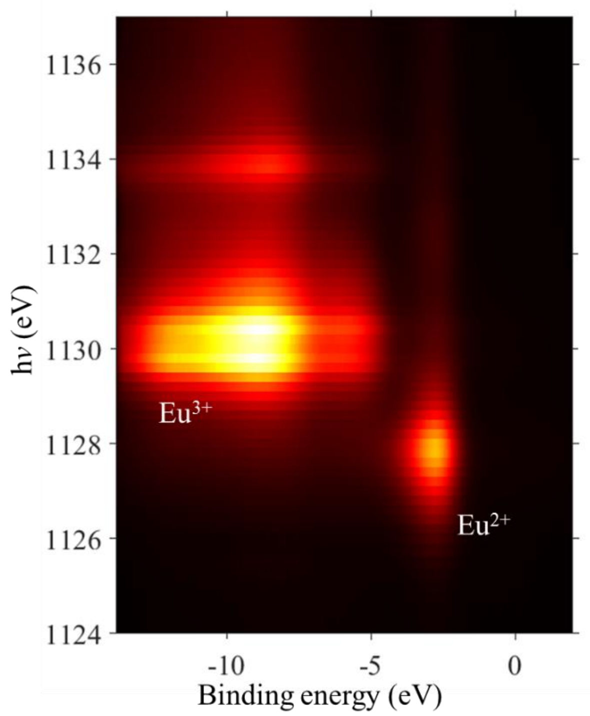

Supplementary Figure S8: Resonance photoemission spectra of valence band near Eu 3d threshold. We see a strong resonance of the Eu^{2+} states in the valence band at h*v* = 1128 eV and of Eu^{3+} states at hv = 1130 eV. No resonating states are seen at the E_F.

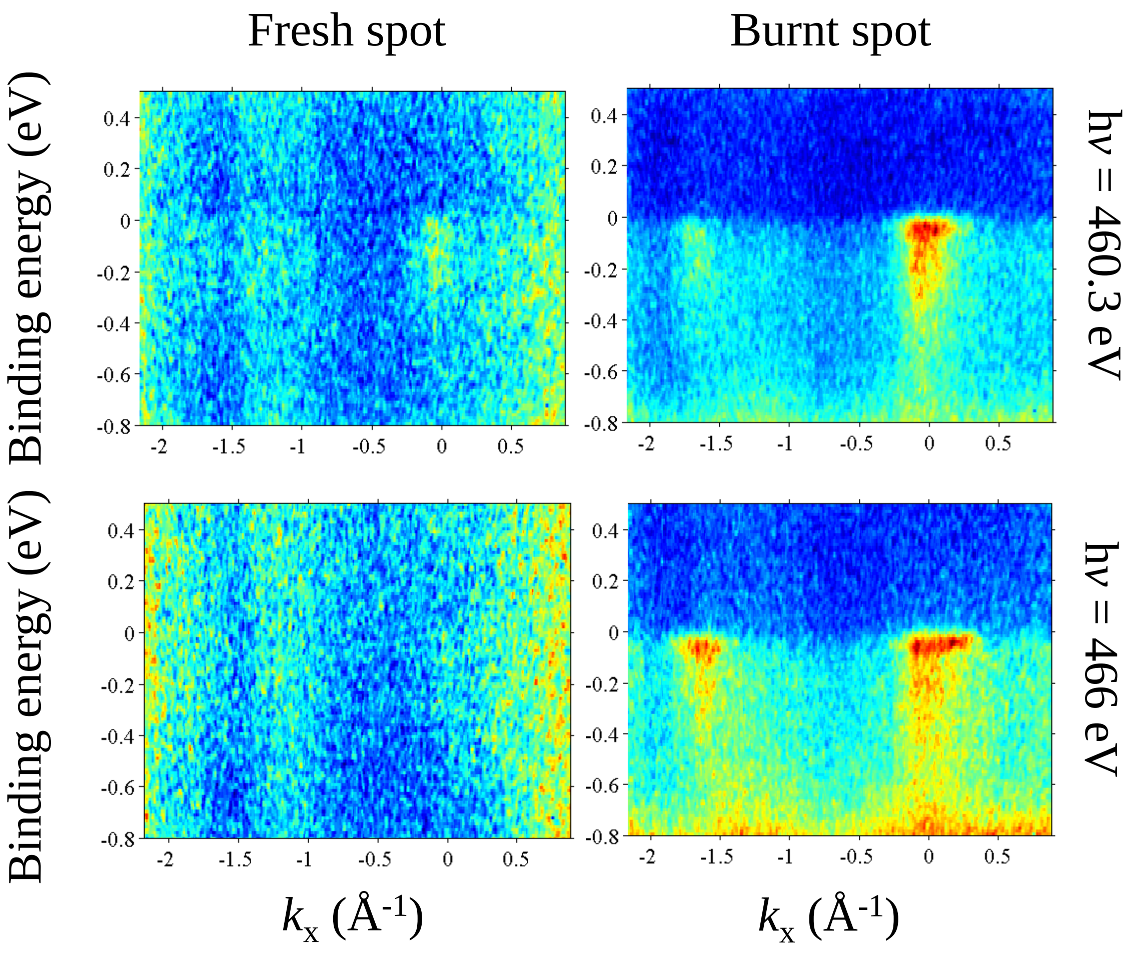

Supplementary Figure S9

2DEG experimental band structure along the ΓX directions measured for *s*-polarization and two photon energies *hv* = 460.3 eV (upper row) and 466 eV probing d_{xy}- and d_{yz}-states (bottom row). Spectra were measured for different X-ray irradiation times: fresh spot; and 'burnt" spot after 30 min irradiation of the sample surface. The SX-ARPES time evolution spectra show the gradual increase of the spectral intensity indicating the sample recovery.

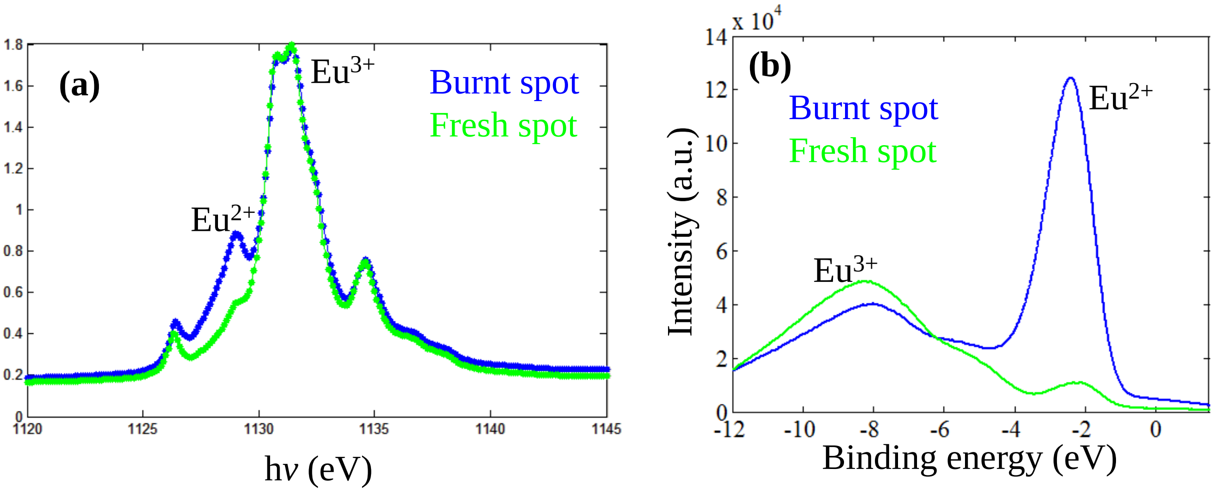

Supplementary Figure S10

(a) Absorption spectra near Eu 4*f* edge. Spectra are measured at 2 spots of the sample surface: fresh spot and "burnt" spot. "Burnt' spot spectrum shows huge increase of the Eu^{2+} ions in comparison to Eu^{3+}. (b) Valence band spectrum measured at h*ν* = 457 eV at "burnt" spot of the sample surface (blue line) shows the prevalence of Eu^{2+} in comparison to the spectrum measured at fresh spot (green line).

Supplementary References

1. Kormondy, K. J. *et al.* Epitaxy of polar semiconductor Co_3O_4 (110): Growth, structure, and characterization. *J. Appl. Phys.* **115,** 243708 (2014).

2. Chambers, S. A., Ohsawa, T., Wang, C. M., Lyubinetsky, I. & Jaffe, J. E. Band offsets at the epitaxial anatase TiO_2/n-$SrTiO_3$ (001) interface. *Surf. Sci.* **603,** 771–780 (2009).

3. Hatch, R. C. & Höchst, H. Evolution of interface properties of the Pentacene/Bi(0001) system. *Surf. Sci.* **604,** 1684–1687 (2010).

4. Chambers, S. A. *et al.* Band discontinuities at epitaxial $SrTiO_3$/Si(001) heterojunctions. *Appl. Phys. Lett.* **77,** 1662 (2000).

5. Onose, Y., Takeshita, N., Terakura, C., Takagi, H. & Tokura, Y. Doping dependence of transport properties in $Fe_{1-x}Co_xSi$. *Phys. Rev. B* **72,** 224431 (2005).

6. Tolsma, J. R., Polini, M. & MacDonald, A. H. Orbital and spin order in oxide two-dimensional electron gases. *Phys. Rev. B* **95,** 205101 (2017).

7. Santander-Syro, A. F. *et al.* Two-dimensional electron gas with universal subbands at the surface of $SrTiO_3$. *Nature* **469,** 189–93 (2011).

8. Ziman.J.M. *Principles of the Theory of Solids*. (1972).

9. Mairoser, T. *et al.* High-quality EuO thin films the easy way via topotactic transformation. *Nat. Commun.* **6,** 7716 (2015).
